# Correction: Effectiveness of PARP Inhibitor Maintenance Therapy in Ovarian Cancer by BRCA1/2 and a Scar-Based HRD Signature in Real-World Practice

**DOI:** 10.1158/1078-0432.CCR-25-2196

**Published:** 2025-08-14

**Authors:** Debra L. Richardson, Julia C.F. Quintanilha, Natalie Danziger, Gerald Li, Ethan Sokol, Alexa B. Schrock, Ericka Ebot, Neeru Bhardwaj, Tanesha Norris, Anosheh Afghahi, Anthony Frachioni, Christina Washington, Lauren Dockery, Julia Elvin, Ryon P. Graf, Kathleen N. Moore

In the original version of this article (1), there was an error in Fig. 4C: 17.1% was labeled as BRCA-WT instead of BRCAalt. The error has been corrected in the latest online and PDF versions of the article. The authors apologize for this error.
